# Prevalence, awareness, treatment, and control of type 2 diabetes mellitus among the adult residents of tehran: Tehran Cohort Study

**DOI:** 10.1186/s12902-022-01161-w

**Published:** 2022-10-17

**Authors:** Alireza Oraii, Akbar Shafiee, Arash Jalali, Farshid Alaeddini, Soheil Saadat, Farzad Masoudkabir, Ali Vasheghani-Farahani, Amirhossein Heidari, Saeed Sadeghian, Mohamamdali Boroumand, Abbasali Karimi, Oscar H. Franco

**Affiliations:** 1 Tehran Heart Center, Cardiovascular Diseases Research Institute, Tehran University of Medical Sciences, Tehran, Iran; 2 Department of Emergency Medicine, University of California, Irvine, California USA; 3 Cardiac Primary Prevention Research Center, Cardiovascular Diseases Research Institute, Tehran University of Medical Sciences, Tehran, Iran; 4 Faculty of Medicine, Tehran Medical Sciences, Islamic Azad University, Tehran, Iran; 5 Institute of Social and Preventive Medicine (ISPM), University of Bern, Bern, Switzerland

**Keywords:** Diabetes mellitus, Prevalence, Awareness, Treatment, Control, Epidemiology

## Abstract

**Background:**

The prevalence of type 2 diabetes mellitus has increased in the past decades. We investigated the prevalence of diabetes and its awareness, treatment, and control among adult residents of Tehran.

**Methods:**

We used the recruitment phase data of the Tehran Cohort study, enrolling a random sample of adult residents of Tehran aged ≥35 years. Diabetes was defined as self-report, current use of glucose-lowering medications, and/or fasting plasma glucose (FPG) ≥126mg/dl. Impaired fasting glucose (IFG) was defined as an FPG of 100-125mg/dl. Awareness was defined as diabetes self-report, treatment as receiving glucose-lowering medications, and glycemic control as FPG <126mg/dl. The age- and sex-weighted estimates were calculated using the 2016 national census. Logistic regression models were used to determine the factors associated with diabetes awareness, treatment, and control.

**Results:**

A total of 8151 participants were included. Age- and sex-weighted prevalence of diabetes mellitus and IFG were 16.7% (95% CI: 15.1–18.4) and 25.1% (95% CI: 23.1–27.1), respectively. Diabetes was more prevalent in the eastern and central districts of Tehran. Advanced age (OR per 1-year increase: 1.026, 95% CI: 1.021-1.030), male sex (OR: 1.716, 95% CI: 1.543-1.909), higher BMI levels (OR for BMI ≥35 vs. <20 kg/m^2^: 4.852, 95% CI: 3.365-6.998), pre-existing hypertension (OR: 1.552, 95% CI: 1.378-1.747), dyslipidemia (OR: 1.692, 95% CI: 1.521-1.883), and chronic kidney disease (OR: 1.650, 95% CI: 1.019-2.673) were associated with an increased odds of diabetes mellitus. On the contrary, diabetes mellitus was less likely in current tobacco (OR: 0.872, 95% CI: 0.765-0.994) and alcohol users (OR: 0.836, 95% CI: 0.703-0.994) compared to non-users. Among diabetic individuals, 82.8% were aware of their condition, 71.9% received treatment, and 31.7% of treated patients had adequate glycemic control. Advanced age and pre-existing comorbidities, including hypertension and dyslipidemia, were associated with higher diabetes awareness and treatment. Furthermore, advanced age, higher levels of education, and female sex were determinants of better glycemic control among treated diabetic participants.

**Conclusion:**

There is a high prevalence of diabetes and IFG among adult residents of Tehran. Additionally, more than two-thirds of treated diabetics living in Tehran remain uncontrolled.

**Supplementary Information:**

The online version contains supplementary material available at 10.1186/s12902-022-01161-w.

## Introduction

Type 2 diabetes mellitus is one of the major global health concerns of the 21^st^ century. The International Diabetes Federation estimates that 9.3% of adults aged 20-79 (463 million people) have diabetes, which is projected to reach 578 million adults by 2030 [1]. The 2017 Global Burden of Diseases studies further estimated that high fasting plasma glucose (FPG) was the third most common global risk factor for disability-adjusted life years [2]. Notably, diabetes mellitus is an important risk factor for cardiovascular diseases [3–5]. This signifies the vital role of up-to-date epidemiological studies in designing national and local health policies.

The age-standardized diabetes prevalence in adults has increased globally over the past decades [6]. The increase in diabetes burden and number of adults with diabetes was more substantial in low- and middle-income countries than in high-income countries. Population aging, urbanization, sedentary lifestyle, and unhealthy dietary habits have been proposed as major drivers of this increasing trend [7]. Similarly, the number of diabetic patients has been rising among the Iranian population for the past 20 years [8]. A recent study showed a prevalence of 15.0% for diabetes mellitus and 25.4% for prediabetes in some provinces of Iran that did not encompass Tehran [9]. However, there is a considerable variation in the prevalence of diabetes between different geographical regions of the country [10–12].

Tehran, the capital of Iran, is a heavily populated metropolis with over eight million individuals. Although there has been an increasing trend of diabetes in Tehran [13], few epidemiological studies have described its prevalence in Tehran. These studies are limited by district-level sampling, specific patient populations, and/or methods of diagnosing diabetes mellitus [14, 15]. Therefore, one cannot judge the accurate prevalence of known cases of diabetes as well as newly diagnosed cases among the adult population of Tehran. Additionally, the rate of awareness, treatment, and glycemic control of people with type 2 diabetes in Tehran remains unclear and requires further investigation.

The lack of up-to-date data regarding the prevalence, awareness, treatment, and glycemic control of diabetes in Tehran warrants a comprehensive epidemiological study on these issues. Such data can be implemented in future health policies and public health interventions to reduce the diabetes burden and associated disabilities. Therefore, we aimed to determine the prevalence of diabetes mellitus type 2 and its awareness, treatment, and control rate among adult residents of Tehran, utilizing data from the Tehran Cohort study (TeCS).

## Methods

### Study design and participants

In the present study, we analyzed data from TeCS, a population-based prospective study of adult residents of Tehran, a heavily-populated metropolitan area in the Middle East. The study protocol of TeCS has been published in detail elsewhere [16]. In brief, a sample of adult residents of Tehran aged ≥35 years were enrolled through a systematic random sampling method. A total of 4215 households comprising 8296 adults aged ≥35 years participated in the study between May 2016 and February 2019. Participants lacking data regarding self-report of a previous diagnosis of diabetes, medication history, and/or FPG, as well as type 1 diabetes mellitus, were excluded from the present study (*n*=145). In the end, data from 8151 participants were included for further analyses. The research deputy and the ethical committee of the Tehran University of Medical Sciences approved the study protocol (IR.TUMS.MEDICINE.REC.1399.074). Additionally, all participants signed a written informed consent before enrollment.

### Data collection and measurements

Data regarding demographic characteristics, pre-existing comorbidities, metabolic and behavioral risk factors, physical activity, and level of education were gathered through detailed in-person interviews. Furthermore, standard anthropometric indices, including weight, height, and waist and hip circumference, were measured in all participants. Body mass index (BMI) was calculated as weight divided by the square of height (kg/m^2^). Waist circumference was measured at the top of the iliac crest at the approximate level of the umbilicus, and hip circumference was taken around the widest portion of the buttocks. A fasting venous blood sample was obtained to measure FPG level after an overnight fast of 8-12 hours.

### Definitions

We used the standard criteria laid by the World Health Organization and International Diabetes Federation for defining type 2 diabetes mellitus and glycemic control [17]. Diabetes mellitus was defined as having any of the following: 1) self-report of a previous diagnosis of type 2 diabetes mellitus by healthcare providers, 2) current use of any glucose-lowering medications including oral hypoglycemic agents or insulin, or 3) an FPG of ≥126 mg/dl after an overnight fast of 8-12 hours. Impaired fasting glucose (IFG) was defined as an FPG of 100 to 125 mg/dl without a previous diagnosis of type 2 diabetes or the current use of glucose-lowering medication. According to the above criteria, participants with abnormal glucose metabolism were defined as having either diabetes mellitus or IFG. Individuals with either self-reported diabetes or current use of glucose-lowering medications were considered as known cases of diabetes. In contrast, those without a self-report of diabetes or medication use in whom a diagnosis of diabetes was made based on our laboratory measurement of FPG were considered new cases of diabetes. Awareness was defined as a self-report of a previous diagnosis of diabetes and treatment as a self-report of receiving any glucose-lowering medication among diabetic participants. Glycemic control was also defined as an FPG of less than 126 mg/dl among diabetic participants under treatment [17].

Pre-existing comorbidities included hypertension, dyslipidemia, or chronic kidney disease, defined as the previous diagnosis of the condition or related medication use. Definitions of tobacco use, opium consumption, and alcohol use were described in our study protocol previously [16]. In brief, current tobacco use was considered daily or occasional cigarette, pipe, or hookah smoking. Former tobacco users were those with a history of smoking who had quit for at least one month before the interview. Opium consumption was defined as any use of opium or its derivatives in the previous year. Alcohol consumption was considered as any use of alcoholic products within the preceding year. Physical activity was categorized as low, intermediate, and high activity based on a Likert-scale questionnaire.

### Statistical analyses

Categorical variables were reported as the frequency with percentage and were compared between groups using the chi-square test or Fisher exact test. Continuous data were expressed as mean with standard deviation or median (interquartile range boundaries) according to the normality distribution of data. An independent student t-test was used to compare continuous data between two groups. Due to different age and sex distribution in our study population compared with the adult population of Tehran, age- and sex-weighted rates were calculated for the prevalence of diabetes and IFG in the overall study population, using Tehran's adult population aged ≥35 years derived from the 2016 national census. Moreover, the age-weighted prevalence of diabetes and IFG were calculated for men and women. The prevalence of diabetes, awareness, treatment, and control among various age, sex, BMI, metabolic risk factor, physical activity, and education subgroups were further analyzed to examine variations of mentioned indices by different subpopulations. We assessed the effect of baseline covariates on the odds of having diabetes mellitus and abnormal glucose metabolism using a generalized logistic regression model for ordinal dependent variables adjusting for age, sex, marital status, level of education, BMI categories, hypertension, dyslipidemia, chronic kidney disease, physical activity level, tobacco use, alcohol, and opium consumption. This model estimates the partial proportional odds assumption, relaxing the parallel lines assumption for all independent variables. Furthermore, we assessed the determinants of awareness, treatment, and glycemic control among diabetic participants using a logistic regression model including all of the abovementioned baseline covariates. The household code was entered in all models to adjust for potential cluster effects. Odds ratio (OR) with an associated 95% confidence interval (CI) was used for reporting the adjusted effects. All statistical analyses were performed using the IBM SPSS Statistics for Windows, v.25.0 (IBM Corp., Armonk, NY, USA) and STATA version 14.2 (College Station, TX: Stata Corp LP.). A *p*-value of <0.05 was considered statistically significant. The Geographical distribution of diabetes, IFG, awareness, treatment, and sufficient control were depicted in the Tehran map using the first three digits of the postal code using *shp2dta* and *spmap* modules in STATA version 14.2 (College Station, TX: Stata Corp LP.).

## Results

Data from 8151 participants were included in the present study. The mean age of the study population was 53.7 ± 12.73 years, and 4420 (54.2%) participants were women. Of the study population, 71.9% had a BMI of ≥25 kg/m2, 17.6% reported low daily physical activity, and 99.2% were married. A detailed description of the total study population characteristics is shown in Supplementary Table 1.

### Prevalence of type 2 diabetes mellitus and impaired fasting glucose

**Fig. 1:**
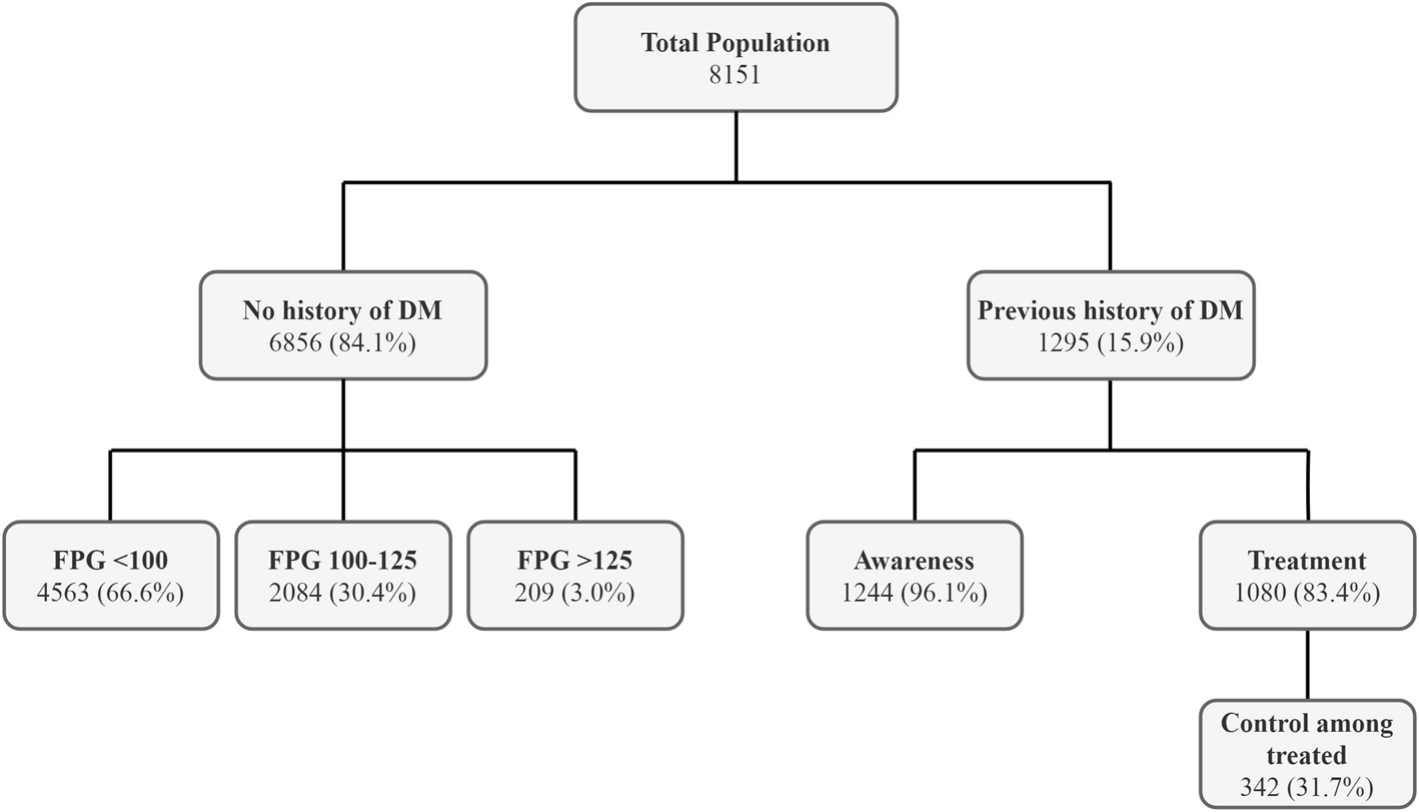
Flowchart of including participants from the Tehran Cohort Study for this analysis

**Fig. 2:**
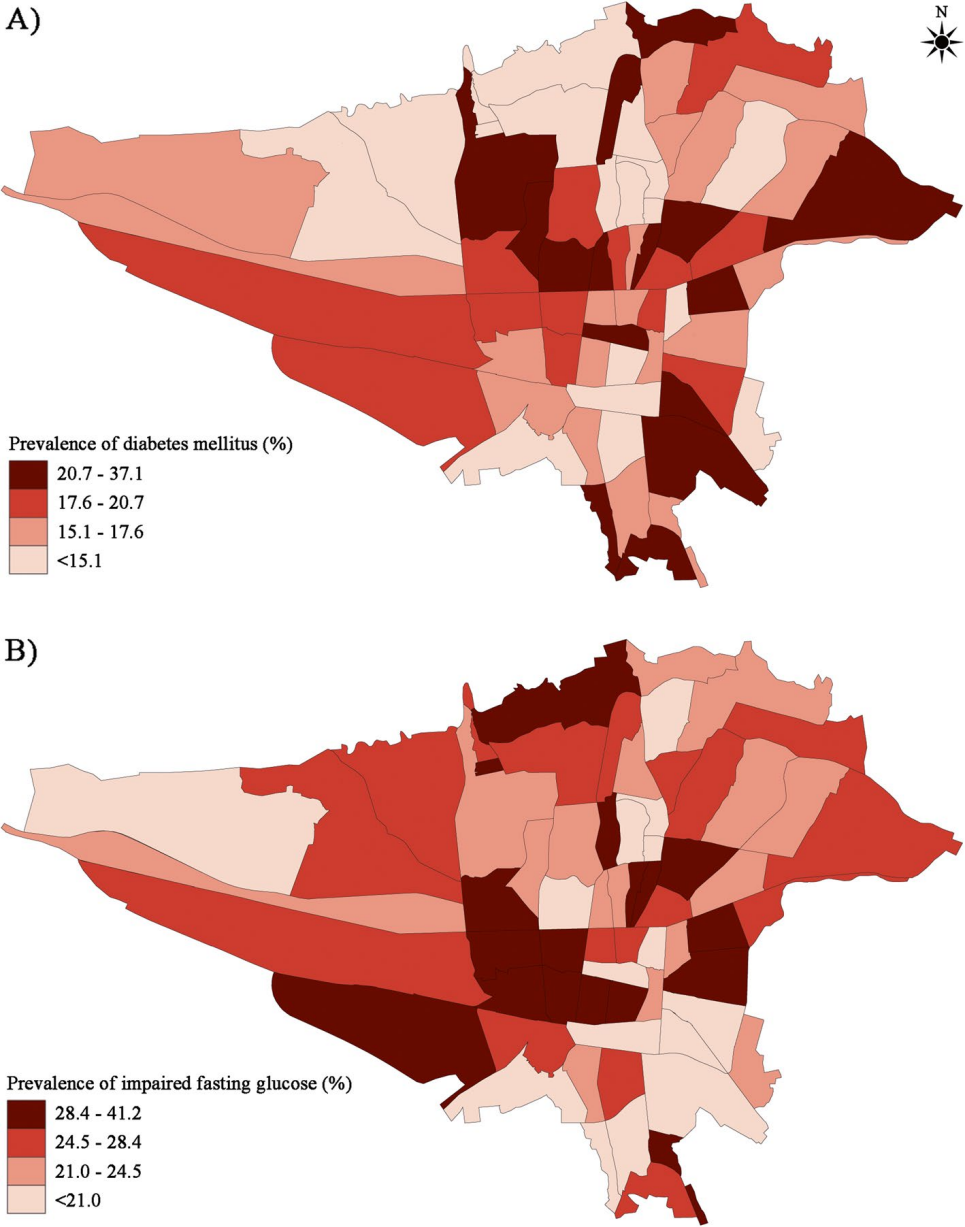
Geographic distribution of the prevalence of (**a**) diabetes mellitus, and (**b**) impaired fasting glucose based on the postal regions of Tehran

The study participants were categorized into diabetes mellitus, IFG, or normal. A total of 1295 (15.9%) participants had a previous diagnosis of type 2 diabetes mellitus or received glucose-lowering medication (Fig. 1). Considering the participants having an FPG ≥126 mg/dl, the number of diabetics increased to 1504 (18.5%). Overall, the calculated age- and sex-weighted prevalence of diabetes was 16.7% (95% CI: 15.1 – 18.4) among adult residents of Tehran aged ≥35 years. The age-weighted prevalence of type 2 diabetes mellitus was 16.8% (95% CI: 14.2 – 19.1) in men and 16.6% (95% CI: 14.6 – 19.2) in women. In addition, a total of 2084 (25.6%) participants had IFG based on the obtained blood samples, which would result in an age- and sex-weighted IFG prevalence of 25.1% (95% CI: 23.1 – 27.1) among adult residents of Tehran aged ≥35 years. The age-weighted prevalence of IFG was 29.3% (95% CI: 26.3 – 32.5) in men and 21.7% (95% CI: 19.1 – 24.4) in women. As depicted in Fig 2, the prevalence of diabetes was higher in the Eastern and Central parts of Tehran, and IFG was more observed in the Central and Northern regions.

**Fig. 3:**
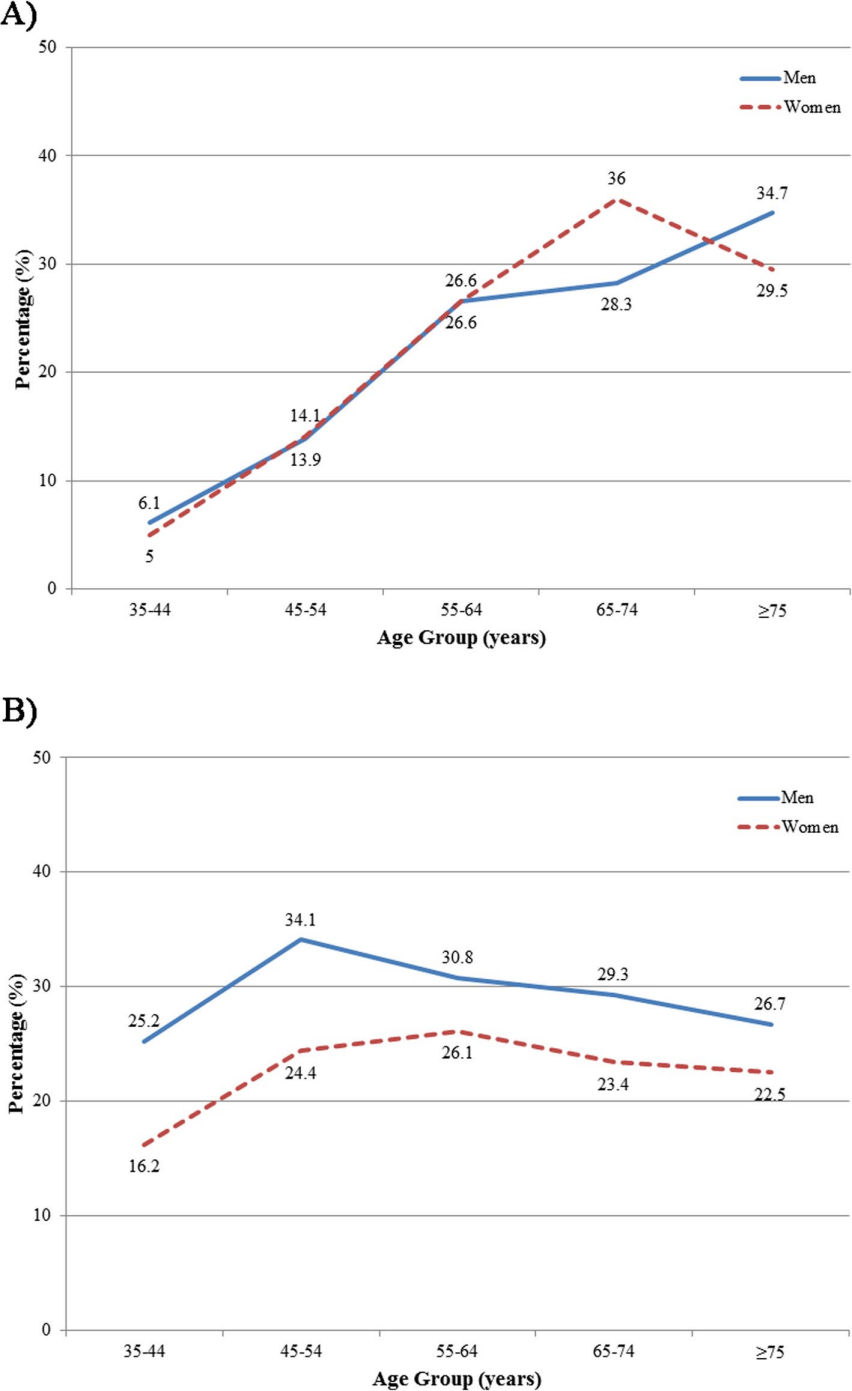
Prevalence of (**a)** diabetes mellitus and (**b)** impaired fasting glucose stratified by age and sex

**Fig. 4:**
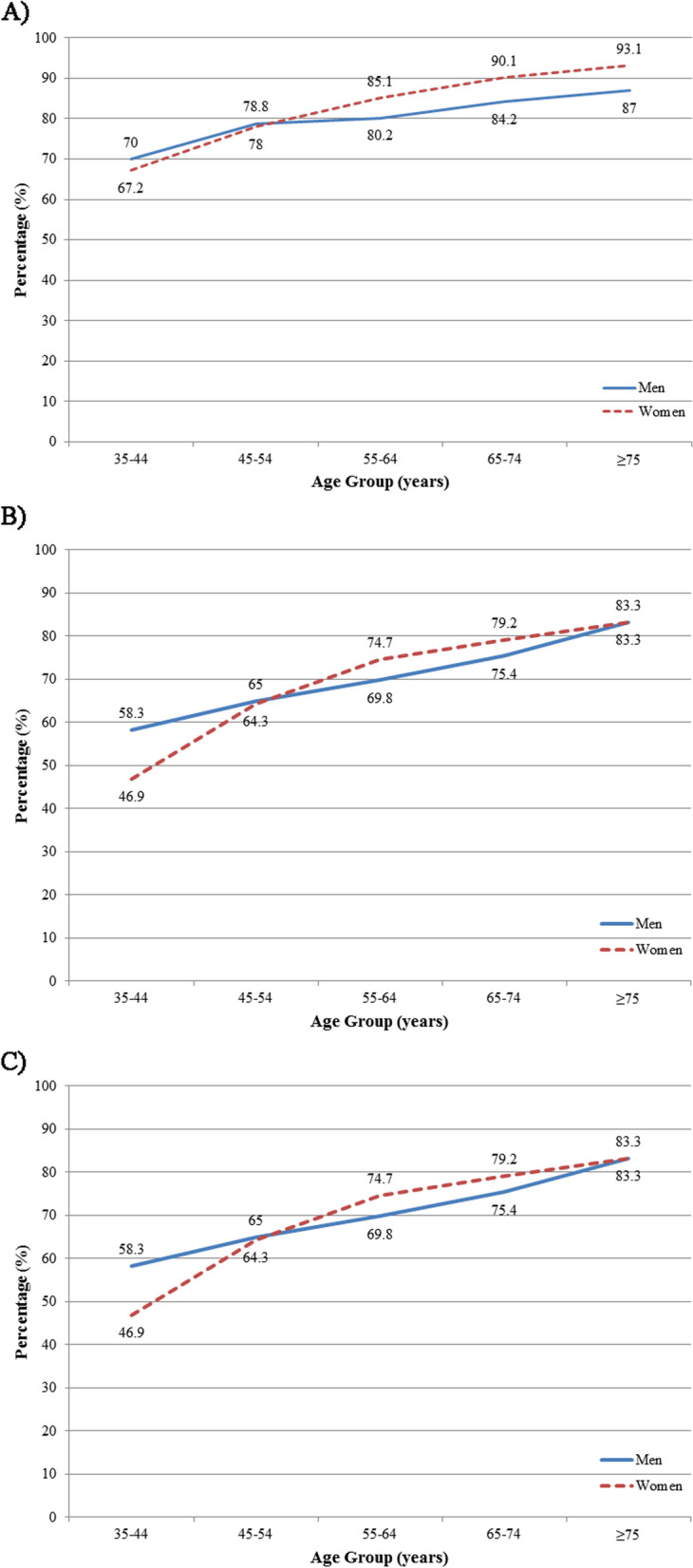
Prevalence of diabetes mellitus (**a**) Awareness, (**b**) Treatment, and (**c**) Control among treated diabetic individuals stratified by age and sex

**Fig. 5:**
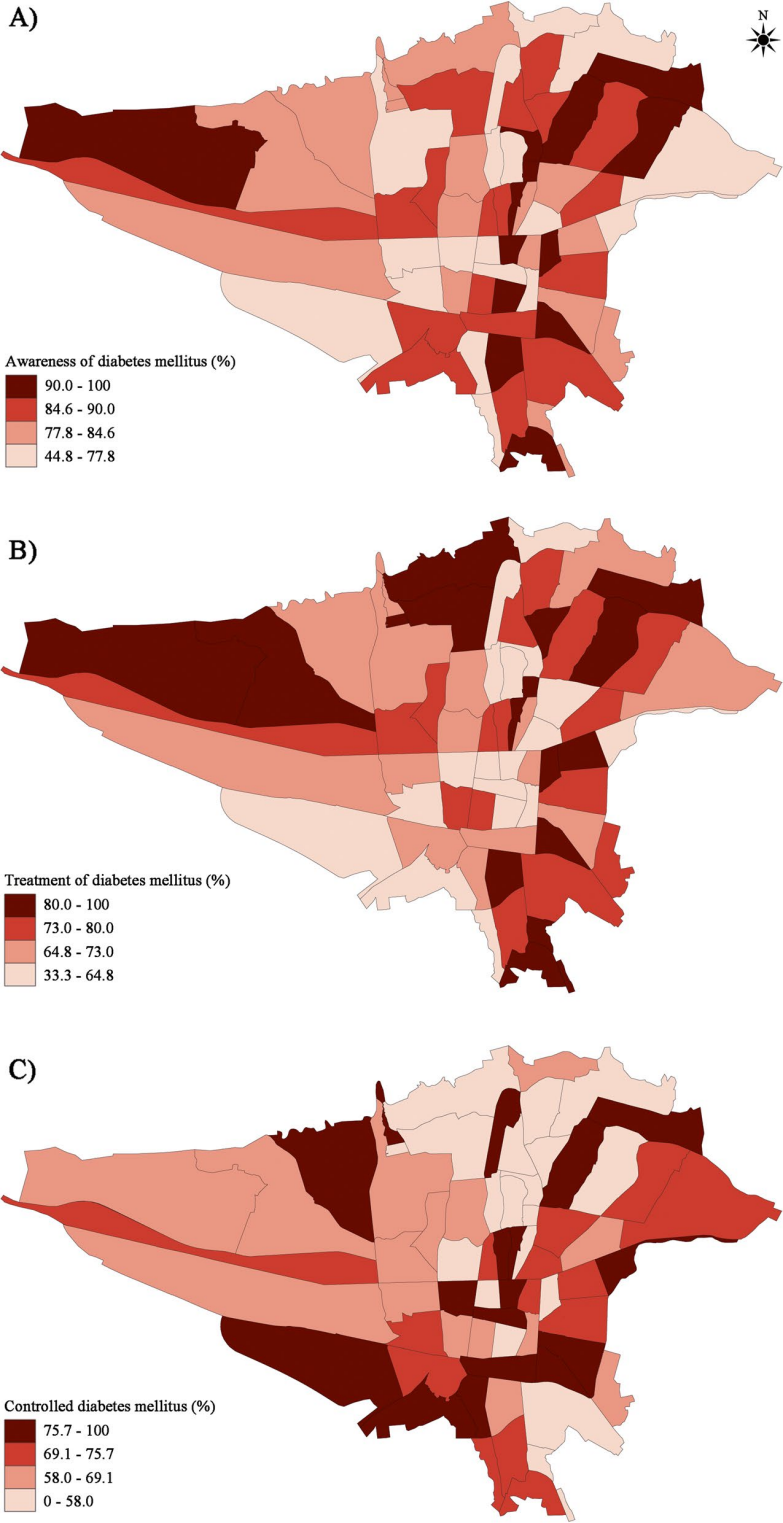
Geographic distribution of diabetes mellitus (**a**) Awareness, (**b**) Treatment, and (**c**) Control among treated diabetic individuals based on the postal regions of Tehran

A detailed descriptive report of the prevalence of diabetes mellitus and IFG across baseline characteristic subgroups is shown in 1. The mean age of diabetic individuals was 60.7 ± 11.33 years, and the prevalence of diabetes increased considerably with advancing age, from 5.4% in individuals aged between 35-44 years to 32.4% in those over 75 years (Supplementary Table 2, Fig. 3a). The mean age of participants with IFG was 54.7 ± 12.03 years, significantly lower than diabetic individuals (*P*-value <0.001). IFG increased with advancing age to peak in participants aged 45-54 years and showed a decreasing trend in the following age categories (Supplementary Table 2, Fig. 3b).

Detailed associations between baseline characteristics and diabetes mellitus are shown in 2. The multivariable-adjusted model showed that advancing age (OR per 1-year increase 1.026, 95% CI: 1.021-1.030) and male sex (OR 1.716, 95% CI: 1.543-1.909) were significantly associated with higher odds of having diabetes mellitus. A higher level of BMI was associated with higher odds for diabetes mellitus (OR for BMI ≥35 vs. <20 kg/m^2^: 4.852, 95% CI: 3.365-6.998). In addition, participants with pre-existing hypertension (OR 1.552, 95% CI: 1.378-1.747), dyslipidemia (OR 1.692, 95% CI: 1.521-1.883), or chronic kidney disease (OR 1.650, 95% CI: 1.019-2.673) were more likely to have diabetes mellitus. On the other hand, We observed lower odds of diabetes mellitus in current tobacco users (OR 0.872, 95% CI: 0.765-0.994) and alcohol users (OR 0.836, 95% CI: 0.703-0.994) compared to non-users. Nevertheless, we did not observe any significant association between diabetes mellitus and level of education, physical activity, and opium consumption.

The associations between baseline characteristics and abnormal glucose metabolism were similar to the abovementioned relationships for diabetes mellitus (2), except for participants with high physical activity levels who had a lower odds of abnormal glucose metabolism (OR 0.678, 95% CI: 0.562-0.819) compared to low active individuals.

### Awareness, treatment, and control of type 2 diabetes mellitus

#### Awareness

We identified 209 cases with an elevated FPG with no history of diabetes mellitus. Meanwhile, 51 participants took glucose-lowering medications without reporting a history of diabetes mellitus. Hence, in the total of 1504 diabetic participants in our study, 1244 (82.8%) diabetic participants were aware of their diabetes status. A descriptive report of diabetes awareness across baseline characteristic subgroups is shown in 3. In our study population, awareness was lowest among younger individuals but significantly improved with advancing age (35-44 years: 68.5% vs. ≥75 years: 89.4%). Awareness was similar in men and women with an established diagnosis of diabetes mellitus (81.1% vs. 84.2%, respectively) (Supplementary Table 3, Fig. 4a).

The adjustment model showed significantly higher odds of diabetes awareness among diabetic participants aged 65-74 years (OR 1.955, 95% CI: 1.116-3.425) and ≥75 years (OR 2.104, 95% CI: 1.043-4.246) compared to those aged 35-45 years. In addition, pre-existing history of hypertension (OR 1.548, 95% CI: 1.120-2.141) and dyslipidemia (OR 2.212, 95% CI: 1.649-2.967) were associated with an improved awareness among diabetic individuals. However, there was no significant association between diabetes awareness and sex, marital status, BMI, pre-existing chronic kidney disease, level of education, physical activity, tobacco use, alcohol, and opium consumption (3). Awareness of diabetes was more frequent among diabetic participants living in the Northeast and Southeast districts of Tehran (Fig. 5a).

### Treatment

In the present study, treatment with glucose-lowering medications was reported in 1080 (71.9%) diabetic participants, which comprised 83.4% of cases with known diabetes. Among treated participants, 865 (80.1%) received oral hypoglycemic agents, 97 (9.0%) received insulin, and 118 (10.9%) received both oral agents and insulin for the treatment of hyperglycemia. A descriptive report of receiving treatment for diabetes across baseline characteristic subgroups is shown in 3. The proportion of individuals receiving glucose-lowering medication increased considerably with advancing age (35-44 years: 52.4% vs. ≥75 years: 83.3%), while no significant difference was observed between men and women (71.3% vs. 72.3%, respectively) (Supplementary Table-3, Fig. 4b).

In the adjusted model, the odds ratio of receiving glucose-lowering medications for the treatment of diabetes was significantly higher among participants aged 55-64 years (OR 1.719, 95% CI: 1.093-2.703), 65-74 years (OR 1.955, 95% CI: 1.196-3.195), and ≥75 years (OR 2.538, 95% CI: 1.380-4.666) compared to younger individuals (3). Pre-existing comorbidities, including hypertension (OR 1.502, 95% CI: 1.159-1.946) and dyslipidemia (OR 1.999, 95% CI: 1.558-2.565), were independent predictors of receiving treatment. However, no significant association was found between receiving treatment and sex, marital status, level of education, BMI level, physical activity, tobacco use, alcohol, and opium consumption (3). Diabetic participants residing in Tehran's Northern and Eastern districts were more likely to receive glucose-lowering treatment (Fig. 5b).

### Control among treated

Among diabetic participants receiving treatment, 342 (31.7%) individuals had adequate fasting glycemic control. Based on the type of treatment, glycemic control was observed among 285 (33.0%) participants receiving oral hypoglycemic agents, 30 (30.9%) participants receiving insulin injections, and 27 (22.9%) participants receiving both insulin and oral agents (*P*-value=0.084). A descriptive report of glycemic control across baseline characteristic subgroups is shown in 3. Diabetes control was poorest among individuals aged 45-54 years (25.9%) but increased with advancing age to reach a peak of 47.0% among those ≥75 years old (Supplementary Table 3, Fig. 4c). In contrast with awareness and treatment, diabetes control was significantly better among women than men (34.5% vs. 28.5%, respectively).

The adjusted model confirmed a significant rise in glycemic control rate in those aged ≥75 years (OR 2.475, 95% CI: 1.213-5.050) and women (OR 1.789, 95% CI: 1.295-2.471) (3). In addition, compared to illiteracy, having >12 years of education (OR 1.919, 95% CI: 1.131-3.256) was significantly associated with higher odds of having adequate glycemic control. There was no significant association between diabetes control and marital status, level of education, BMI level, pre-existing comorbidities, physical activity, tobacco use, alcohol, and opium consumption (Table 3). Unlike the treatment pattern, diabetic residents in the Northern districts of Tehran had a lower frequency of diabetes control (Fig. 5c).

**Table 1:** Baseline characteristics and prevalence of diabetes mellitus in the population of Tehran Cohort Study

	**Normal**^a^ ***n*=4563**	**Impaired Fasting Glucose**^a^ ***n*=2084**	**Diabetes Mellitus**^a^ ***n*=1504**	***p-*value **^**‡**^
**Age, mean ± SD, year**	50.9 ± 12.51	54.7 ± 12.03	60.7 ± 11.33	<0.001
**Age, year, n (%)**				<0.001
35-44	1715 (74.5)	463 (20.1)	125 (5.4)	
45-54	1245 (57.2)	627 (28.8)	305 (14.0)	
55-64	870 (45.3)	541 (28.1)	511 (26.6)	
65-74	496 (41.5)	315 (26.4)	383 (32.1)	
≥75	237 (42.7)	138 (24.9)	180 (32.4)	
**Sex, n (%)**				<0.001
Men	1929 (51.7)	1104 (29.6)	698 (18.7)	
Women	2634 (59.6)	980 (22.2)	806 (18.2)	
**Marital status, n (%)**				0.786
Married	4511 (55.9)	2066 (25.6)	1489 (18.5)	
Non-married	37 (56.1)	15 (22.7)	14 (21.2)	
**Education, year, n (%)**				<0.001
Illiterate	271 (47.4)	120 (21.0)	181 (31.6)	
1-5	389 (47.2)	221 (26.8)	215 (26.1)	
6-12	2304 (54.5)	1128 (26.7)	799 (18.9)	
>12	1581 (63.2)	612 (24.5)	307 (12.3)	
**Waist circumference, mean ± SD, cm**	93.6 ±11.26	98.6 ± 11.37	101.2 ±11.63	<0.001
**Hip circumference, mean ± SD, cm**	103.9 ±9.47	106.3 ±9.72	107.6 ±10.84	<0.001
**Body mass index, kg/m**^**2**^**, n (%)**				<0.001
<20	171 (77.0)	39 (17.6)	12 (5.4)	
20-24.9	1361 (66.6)	420 (20.5)	263 (12.9)	
25-29.9	1886 (55.9)	898 (26.6)	589 (17.5)	
30-34.9	843 (47.8)	512 (29.0)	408 (23.1)	
≥35	265 (39.6)	197 (29.4)	207 (30.9)	
**Hypertension, n (%)**				<0.001
No	3685 (63.0)	1486 (25.4)	674 (11.5)	
Yes	863 (37.8)	594 (26.0)	829 (36.3)	
**Dyslipidemia, n (%)**				<0.001
No	3456 (63.1)	1439 (26.3)	584 (10.7)	
Yes	1091 (41.2)	641 (24.2)	919 (34.7)	
**Chronic kidney disease, n (%)**				<0.001
No	4543 (56.2)	2069 (25.6)	1468 (18.2)	
Yes	20 (28.2)	15 (21.1)	36 (50.7)	
**Tobacco user, n (%)**				<0.001
Current	930 (59.1)	409 (26.0)	234 (14.9)	
Former	139 (43.0)	94 (29.1)	90 (27.9)	
Never	3476 (55.8)	1576 (25.3)	1179 (18.9)	
**Opium consumption, n (%)**				0.566
No	4304 (56.1)	1956 (25.5)	1415 (18.4)	
Yes	229 (53.5)	117 (27.3)	82 (19.2)	
**Alcohol consumption, n (%)**				0.008
No	4091 (55.6)	1882 (25.6)	1391 (18.9)	
Yes	441 (60.2)	185 (25.3)	106 (14.5)	
**Physical activity, n (%)**				<0.001
Low	678 (47.6)	355 (24.9)	391 (27.5)	
Intermediate	2636 (56.3)	1174 (25.1)	869 (18.6)	
High	1209 (61.4)	530 (26.9)	229 (11.6)	

*Abbreviations*: *SD* Standard deviation

^**a**^Percentages are calculated in rows

^**‡**^ *P*-value is calculated between normal, impaired fasting glucose, and diabetes subgroups

**Table 2:** Determinants of diabetes mellitus and abnormal glucose metabolism in the Tehran Cohort Study

	**Diabetes Mellitus**		**Abnormal Glucose Metabolism (Diabetes Mellitus + IFG)**	
	**Adjusted OR (95% CI)**	***p-*value**	**Adjusted OR (95% CI)**	***p-*value**
**Age, per 1-year increase**	1.026 (1.021-1.030)	<0.001	1.035 (1.029-1.041)	<0.001
**Male sex**	1.716 (1.543-1.909)	<0.001	1.411 (1.236-1.609)	<0.001
**Married**	0.953 (0.566-1.604)	0.858	0.953 (0.566-1.604)	0.858
**Education, year**				
Illiterate	Ref.		Ref.	
1-5	1.226 (0.987-1.523)	0.064	1.226 (0.987-1.523)	0.064
6-12	1.218 (1.010-1.468)	0.039	1.218 (1.010-1.468)	0.039
>12	0.932 (0.761-1.140)	0.496	0.932 (0.761-1.140)	0.496
**Body mass index, kg/m**^**2**^				
<20	Ref.		Ref.	
20-24.9	1.730 (1.226-2.440)	0.002	2.143 (1.493-3.077)	<0.001
25-29.9	2.543 (1.813-3.566)	<0.001	2.543 (1.813-3.566)	<0.001
30-34.9	3.313 (2.346-4.679)	<0.001	3.313 (2.346-4.679)	<0.001
≥35	4.853 (3.365-6.998)	<0.001	4.853 (3.365-6.998)	<0.001
**Hypertension**	1.552 (1.378-1.747)	<0.001	1.918 (1.671-2.201)	<0.001
**Dyslipidemia**	1.692 (1.521-1.883)	<0.001	2.826 (2.483-3.216)	<0.001
**Chronic kidney disease**	1.650 (1.019-2.673)	0.042	1.650 (1.019-2.673)	0.042
**Tobacco user**				
Never	Ref.		Ref.	
Former	1.117 (0.886-1.410)	0.347	1.117 (0.886-1.410)	0.347
Current	0.872 (0.765-0.994)	0.041	0.872 (0.765-0.994)	0.041
**Opium consumption**	0.936 (0.757-1.158)	0.545	0.936 (0.757-1.158)	0.545
**Alcohol consumption**	0.836 (0.703-0.994)	0.043	0.836 (0.703-0.994)	0.043
**Physical activity**				
Low	Ref.		Ref.	
Intermediate	0.943 (0.833-1.067)	0.358	0.943 (0.833-1.067)	0.358
High	0.894 (0.769-1.039)	0.147	0.678 (0.562-0.819)	<0.001

*Abbreviations*: *OR* Odds ratio, *CI* Confidence interval

All variables are included in the multivariable logistic regression model for calculating the adjusted odds ratios

**Table 3:** Determinants of diabetes awareness, treatment, and glycemic control among the diabetic population of Tehran Cohort Study

	**Awareness**^**a**^ *n*=1244 (82.8%)			**Treatment**^**a**^ *n*=1080 (71.9%)			**Control among treated**^**a**^ *n*=342 (31.7%)		
	**n (%)**	**Adjusted OR (95% CI)**	***p-*value**	**n (%)**	**Adjusted OR (95% CI)**	***p-*value**	**n (%)**	**Adjusted OR (95% CI)**	***p-*value**
**Age, year**									
35-44	85 (68.5)	Ref.		65 (52.4)	Ref.		22 (33.8)	Ref.	
45-54	239 (78.4)	1.448 (0.871-2.407)	0.153	197 (64.6)	1.409 (0.890-2.229)	0.143	51 (25.9)	0.760 (0.394-1.469)	0.416
55-64	424 (83.0)	1.491 (0.908-2.446)	0.114	371 (72.6)	1.719 (1.093-2.703)	0.019	99 (26.8)	0.812 (0.430-1.535)	0.523
65-74	335 (87.5)	1.955 (1.116-3.425)	0.019	297 (77.5)	1.955 (1.196-3.195)	0.007	100 (33.7)	1.167 (0.605-2.249)	0.645
≥75	161 (89.4)	2.104 (1.043-4.246)	0.038	150 (83.3)	2.538 (1.380-4.666)	0.003	70 (47.0)	2.475 (1.213-5.050)	0.013
**Sex**									
Men	566 (81.1)	Ref.		498 (71.3)	Ref.		142 (28.5)	Ref.	
Women	678 (84.2)	1.137 (0.811-1.594)	0.455	582 (72.3)	0.878 (0.664-1.161)	0.363	200 (34.5)	1.789 (1.295-2.471)	<0.001
**Marital status**									
Non-married	13 (92.9)	Ref.		11 (78.6)	Ref.		3 (27.3)	Ref.	
Married	1231 (82.7)	0.402 (0.046-3.500)	0.410	1069 (71.8)	0.499 (0.111-2.229)	0.363	339 (31.8)	1.233 (0.312-4.867)	0.764
**Education, year**									
Illiterate	162 (89.5)	Ref.		147 (81.2)	Ref.		46 (31.5)	Ref.	
1-5	184 (85.6)	1.083 (0.571-2.051)	0.806	159 (74.0)	0.867 (0.518-1.450)	0.587	54 (34.0)	1.372 (0.823-2.288)	0.225
6-12	645 (80.7)	0.788 (0.458-1.356)	0.390	554 (69.3)	0.727 (0.468-1.129)	0.157	162 (29.3)	1.230 (0.781-1.936)	0.371
>12	253 (82.4)	1.046 (0.565-1.936)	0.884	220 (71.7)	0.906 (0.546-1.505)	0.704	80 (36.4)	1.919 (1.131-3.256)	0.016
**Body mass index, kg/m**^**2**^									
<20	11 (91.7)	Ref.		10 (83.3)	Ref.		5 (50.0)	Ref.	
20-24.9	230 (87.5)	0.487 (0.053-4.405)	0.522	203 (77.2)	0.483 (0.090-2.581)	0.395	56 (27.6)	0.413 (0.103-1.650)	0.211
25-29.9	489 (83.0)	0.316 (0.035-2.806)	0.302	419 (71.1)	0.333 (0.063-1.743)	0.193	129 (30.9)	0.489 (0.125-1.910)	0.304
30-34.9	331 (81.1)	0.258 (0.029-2.304)	0.226	285 (69.9)	0.318 (0.060-1.671)	0.176	102 (35.8)	0.618 (0.155-2.457)	0.495
≥35	161 (77.8)	1.952 (0.021-1.778)	0.147	145 (70.0)	0.308 (0.057-1.655)	0.170	40 (27.6)	0.361 (0.088-1.480)	0.157
**Hypertension**									
No	516 (76.6)	Ref.		427 (63.4)	Ref.		131 (30.8)	Ref.	
Yes	728 (87.8)	1.548 (1.120-2.141)	0.008	653 (78.8)	1.502 (1.159-1.946)	0.002	211 (32.4)	0.832 (0.611-1.133)	0.245
**Dyslipidemia**									
No	433 (74.1)	Ref.		360 (61.6)	Ref.		107 (29.7)	Ref.	
Yes	811 (88.2)	2.212 (1.649-2.967)	<0.001	720 (78.3)	1.999 (1.558-2.565)	<0.001	235 (32.7)	1.239 (0.912-1.683)	0.170
**Chronic kidney disease**									
No	1209 (82.4)	Ref.		1048 (71.4)	Ref.		328 (31.4)	Ref.	
Yes	35 (97.2)	4.494 (0.657-30.733)	0.125	32 (88.9)	1.859 (0.604-5.722)	0.280	14 (43.8)	1.297 (0.541-3.108)	0.558
**Tobacco user**									
Current	187 (79.9)	Ref.		158 (67.5)	Ref.		55 (35.0)	Ref.	
Former	75 (83.3)	0.914 (0.481-1.736)	0.784	67 (74.4)	0.946 (0.548-1.631)	0.842	21 (31.3)	1.089 (0.602-1.973)	0.776
Never	982 (83.3)	1.119 (0.729-1.717)	0.607	855 (72.5)	0.999 (0.697-1.433)	0.999	266 (31.1)	1.439 (0.940-2.203)	0.094
**Opium consumption**									
No	1172 (82.8)	Ref.		1022 (72.2)	Ref.		326 (32.0)	Ref.	
Yes	68 (82.9)	1.117 (0.563-2.215)	0.751	54 (65.9)	0.693 (0.405-1.186)	0.181	15 (27.8)	0.896 (0.468-1.715)	0.741
**Alcohol consumption**									
No	1160 (83.4)	Ref.		1007 (72.4)	Ref.		314 (31.2)	Ref.	
Yes	79 (74.5)	0.608 (0.349-1.058)	0.079	68 (64.2)	0.762 (0.467-1.244)	0.278	25 (36.8)	1.346 (0.763-2.376)	0.305
**Physical activity**									
Low	341 (87.2)	Ref.		307 (78.5)	Ref.		114 (37.3)	Ref.	
Intermediate	712 (81.9)	0.860 (0.587-1.260)	0.440	615 (70.8)	0.887 (0.654-1.203)	0.442	179 (29.2)	0.833 (0.599-1.157)	0.276
High	181 (79.0)	0.745 (0.453-1.223)	0.245	151 (65.9)	0.705 (0.471-1.055)	0.089	47 (31.1)	0.906 (0.572-1.435)	0.674

*Abbreviations*: OR, odds ratio; CI, confidence interval

^a^Percentages are calculated in rows

All variables are included in the multivariable logistic regression model for calculating the adjusted odds ratios

## Discussion

Based on the data from TeCS, the weighted prevalence of type 2 diabetes was approximately 16.7% among adult residents of Tehran (estimated 700,000 among 4,229,759 individuals), while approximately 25.1% had IFG (estimated 1,000,000 among 4,229,759 individuals). Furthermore, awareness, treatment, and glycemic control among treated individuals in our study population were 82.8%, 71.9%, and 31.7%, respectively.

The global prevalence of type 2 diabetes has increased over the past decades. Although the prevalence of diabetes is much lower in Asia than in Europe and North America, Middle Eastern countries such as Iran and Saudi Arabia are considered additional hot spots of the global diabetes epidemic [18, 19]. Previous population-based studies have revealed a diabetes prevalence of 7.9% to 10.6% among Iranian adults aged 25-64 years [8, 20]. Trend analysis of these studies showed a 35% increase in diabetes prevalence [8]. However, there is wide regional variability in the country. According to the 2016 STEPwise approach to surveillance (STEPS) study in Iran, Tehran province was one of the provinces with a higher prevalence of diabetes [21]. The prevalence of diabetes was substantially higher in Tehran metropolis, a heavily-populated industrialized city within Tehran province, with diabetes being reported in 16.2% and 11.4% of men and women aged ≥25 years, respectively [21]. Thus, Tehran is one of the hot spots of diabetes in Iran, and stringent prevention strategies should be implemented to prevent its burden.

Consistent with previous studies, advancing age, male sex, and higher BMI levels were associated with an increased risk of diabetes [22–24]. Also, the prevalence of type 2 diabetes was considerably higher in participants with pre-existing comorbidities, which might be due to several mutual risk factors of metabolic diseases. Previous studies also showed a higher incidence of diabetes among individuals with low education levels [25, 26]. Despite a higher percentage of diabetes among illiterate participants in our study, high education level was not an independent protective factor against diabetes in the adjusted model. The observed educational inequality in diabetes could be partially explained by being more overweight/obese among those with lower levels of education [25, 27].

Approximately a quarter of adult residents of Tehran aged ≥35 years had IFG, and participants aged 45-54 years had the highest proportion of IFG in our study. These individuals are at increased risk of future development of diabetes, and the high proportion of young individuals with IFG is an alarm for a higher risk of mortality and vascular disease [4]. Although IFG was higher among men across all age groups, the difference in the percentage of individuals with IFG between the two sexes gradually decreased among participants aged ≥55.

Awareness and treatment were relatively high among adult residents of Tehran. While a higher percentage of younger individuals were unaware of their condition and did not receive treatment than the elderly, these indices did not significantly differ between men and women. However, in previous studies, women had a higher diabetes awareness than men [28, 29]. Also, there was a trend toward higher awareness and treatment among women over 55 compared to their male peers. We hypothesize that women, particularly after menopause, might be more sensitive to their health and physical condition than younger men. In addition, those with pre-existing comorbidities were more likely to be aware of their status, receive glucose-lowering medicines, and have more frequent health care visits.

The importance of adequate glycemic control in preventing microvascular complications of diabetes is widely-known. However, a recent study in Iran showed that only 13.2% of these patients had controlled hyperglycemia [30]. Approximately half of these patients had at least one diabetes-related complication, and a quarter had ischemic heart disease. These low glycemic control rates are not exclusive to developing societies since similar unfavorable rates are also observed in high-income countries [31]. Our findings accentuate the global importance of diabetes management in preventing disease burden.

Glycemic control was observed among less than one-third of the study population. Those receiving simultaneous insulin and oral hypoglycemic agents for the treatment of diabetes had relatively worse glycemic control, indicating a more aggressive nature of the disease. A previous study reported that diabetic patients on more intensive treatment regimens had higher mean hemoglobin A1c (HbA1C) levels [32]. Diabetes also seems more uncontrolled among men and younger individuals [5, 33]. While there is a hypothesis that early-onset type 2 diabetes might present with a more aggressive disease course, others believe younger patients probably face more difficulties adhering to a healthy lifestyle, diet, and medications than the elderly [34, 35]. Future studies should focus on more efficient and modern strategies for diabetes control [36, 37].

The lack of association between physical activity and glycemic control contrasts with the common assumption that physical activity could improve diabetes control. Although our assessment by a Likert-scale questionnaire might have biased these results, further analysis of physical activity showed that participants with low activity levels were considerably older (low active: 70.4±11.88 vs. high active: 59.5±10.60 years). It also included a higher percentage of women (low active: 64.9% vs. high active: 44.7%) than those with higher physical activity. Better diabetes control in more senior and female participants could explain why the control rate was better among those with lower physical activity. Nevertheless, a detailed assessment of physical activity in the ongoing follow-up phase of the TeCS will provide more comprehensive data.

Traditionally, the Northern half of Tehran is said to have a higher socioeconomic status than the lower half. Our data showed a higher frequency of diabetes mellitus in Tehran's eastern and central districts. Even with the higher treatment rate, the frequency of individuals with controlled diabetes was lowest in the Northern regions. So, a public health study on the social determinants of health for diabetes in Tehran is necessary.

### Strengths and limitations

This study has several key strengths as it was the first to investigate the epidemiology of diabetes mellitus in a large representative sample from all geographical districts of Tehran. However, this was a cross-sectional analysis and therefore has some innate limitations. We lacked data on postprandial glucose or HbA1c measurements, which could have influenced our results. HbA1c is being measured in our follow-up phase and can provide further insights. Furthermore, the diagnosis of new cases of diabetes in our study was based on a single-session FPG measurement, which might have led to potential misclassifications.

## Conclusion

We observed a high prevalence of type 2 diabetes mellitus and IFG among the adult residents of Tehran. Despite relatively acceptable awareness and treatment rates, the high percentage of poor glycemic control might indicate a high burden of diabetes and its complications in Tehran. Public health interventions and integrated management plans for earlier diagnosis, treatment, and better control of diabetes in Tehran are required.

## Supplementary Information

**Additional file 1:** **Table S 1. **Baseline characteristics of the Tehran Cohort Study participants. **Table S 2. **Prevalence of impaired fasting glucose and diabetes mellitus in adult residents of Tehran. **Table S 3. **Diabetes awareness, treatment, and glycemic control among treated in adult residents of Tehran.

## Data Availability

The datasets generated and/or analyzed during the current study are not publicly available due to the current policy of the Tehran Cohort Study but are available from the corresponding author on reasonable request.

## Abbreviations

BMI: Body mass index
CI: Confidence interval
FPG: Fasting plasma glucose
IFG: Impaired fasting glucose
OR: Odds ratio
