# Supplementary material for: Prevalence, awareness, treatment, and control of type 2 diabetes mellitus among the adult residents of tehran: Tehran Cohort Study
Source: BMC Endocr Disord. 2022 Oct 17;22:248. doi: 10.1186/s12902-022-01161-w (PMC9578278; doi:10.1186/s12902-022-01161-w)
Supplement: Supplementary file 1 — Additional file 1: Table S 1. Baseline characteristics of the Tehran Cohort Study participants. Table S 2. Prevalence of impaired fasting glucose and diabetes mellitus in adult residents of Tehran. Table S 3. Diabetes awareness, treatment, and glycemic control among treated in adult residents of Tehran. [file 12902_2022_1161_MOESM1_ESM.docx]

**SUPPLEMENTARY DATA**

| **Table S1. Baseline characteristics of the Tehran Cohort Study participants** | |
| --- | --- |
|  | **Total population**  **n = 8151** |
| **Age, mean ± SD, year** | 53.7 ± 12.73 |
| **Age, year, n (%)** |  |
| 35-44 | 2303 (28.3) |
| 45-54 | 2177 (26.7) |
| 55-64 | 1922 (23.6) |
| 65-74 | 1194 (14.6) |
| ≥75 | 555 (6.8) |
| **Sex, n (%)** |  |
| Men | 3731 (45.8) |
| Women | 4420 (54.2) |
| **Marital status, n (%)** |  |
| Married | 8066 (99.2) |
| Non-married | 66 (0.8) |
| **Education, year, n (%)** |  |
| Illiterate | 572 (7.0) |
| 1-5 | 825 (10.2) |
| 6-12 | 4231 (52.1) |
| >12 | 2500 (30.8) |
| **Waist** **circumference, mean ± SD, cm** | 96.3 ± 11.78 |
| **Hip circumference, mean ± SD, cm** | 105.2 ± 9.91 |
| **Body mass index, kg/m^2^, n (%)** |  |
| <20 | 222 (2.8) |
| 20-24.9 | 2044 (25.3) |
| 25-29.9 | 3373 (41.8) |
| 30-34.9 | 1763 (21.8) |
| ≥35 | 669 (8.3) |
| **Hypertension, n (%)** |  |
| No | 5845 (71.9) |
| Yes | 2286 (28.1) |
| **Dyslipidemia,** **n (%)** |  |
| No | 5479 (67.4) |
| Yes | 2651 (32.6) |
| **Chronic kidney disease, n (%)** |  |
| No | 8080 (99.1) |
| Yes | 71 (0.9) |
| **Tobacco user, n (%)** |  |
| Current | 1573 (19.4) |
| Former | 323 (4.0) |
| Never | 6231 (76.7) |
| **Opium consumption, n (%)** |  |
| No | 7675 (94.7) |
| Yes | 428 (5.3) |
| **Alcohol consumption, n (%)** |  |
| No | 7364 (91.0) |
| Yes | 732 (9.0) |
| **Physical activity, n (%)** |  |
| Low | 1424 (17.6) |
| Intermediate | 4679 (58.0) |
| High | 1968 (24.4) |

| **Table S2. Prevalence of impaired fasting glucose and diabetes mellitus in adult residents of Tehran** | | |
| --- | --- | --- |
|  | **Men *** | **Women *** |
| **Impaired fasting glucose** | | |
| Age category, year, n (%) | | |
| 35-44 | 250 (25.2) | 213 (16.2) |
| 45-54 | 337 (34.1) | 290 (24.4) |
| 55-64 | 257 (30.8) | 284 (26.1) |
| 65-74 | 177 (29.3) | 138 (23.4) |
| ≥75 | 83 (26.7) | 55 (22.5) |
| **Diabetes Mellitus** | | |
| Age category, year, n (%) | | |
| 35-44 | 60 (6.1) | 65 (5.0) |
| 45-54 | 137 (13.9) | 168 (14.1) |
| 55-64 | 222 (26.6) | 289 (26.6) |
| 65-74 | 171 (28.3) | 212 (36.0) |
| ≥75 | 108 (34.7) | 72 (29.5) |
| * Percentages are calculated in rows. | | |

| **Table S3. Diabetes awareness, treatment, and glycemic control among treated in adult residents of Tehran** | | |
| --- | --- | --- |
|  | **Men *** | **Women *** |
| **Awareness** | | |
| Age category, year, n (%) | | |
| 35-44 | 42 (70.0) | 43 (67.2) |
| 45-54 | 108 (78.8) | 131 (78.0) |
| 55-64 | 178 (80.2) | 246 (85.1) |
| 65-74 | 144 (84.2) | 191 (90.1) |
| ≥75 | 94 (87.0) | 67 (93.1) |
| **Treatment** | | |
| Age category, year, n (%) | | |
| 35-44 | 35 (58.3) | 30 (46.9) |
| 45-54 | 89 (65.0) | 108 (64.3) |
| 55-64 | 155 (69.8) | 216 (74.7) |
| 65-74 | 129 (75.4) | 168 (79.2) |
| ≥75 | 90 (83.3) | 60 (83.3) |
| **Control among treated** | | |
| Age category, year, n (%) | | |
| 35-44 | 12 (34.3) | 10 (33.3) |
| 45-54 | 22 (24.7) | 29 (26.9) |
| 55-64 | 28 (18.1) | 71 (33.0) |
| 65-74 | 41 (31.8) | 59 (35.1) |
| ≥75 | 39 (43.3) | 31 (52.5) |
| * Percentages are calculated in rows. | | |
